# Do good things really come to those who wait? An analysis of the average time of acceptance in Brazilian surgery journals

**DOI:** 10.1590/acb393824

**Published:** 2024-07-22

**Authors:** Lívia Guerreiro de Barros Bentes, Maria Fernanda de Almeida Cavalcante Aranha, Mariana Kondo Obara, Larissa Yoshie Shibata, Pablo Rodrigues Nunes de Souza, José Felipe Teixeira Borges, Leonardo Barbosa Duarte, Luiz Felipe Silva Marcião, Rui Sérgio Monteiro de Barros

**Affiliations:** 1 Universidade do Estado do Pará – Faculdade de Medicina – Laboratório de Cirurgia Experimental – Belém (PA) – Brazil.; 2 Universidade Federal do Pará – Faculdade de Medicina – Laboratório de Cirurgia Experimental – Belém (PA) – Brazil.; 3 Centro Universitário do Estado do Pará – Faculdade de Medicina – Laboratório de Cirurgia Experimental – Belém (PA) – Brazil.

**Keywords:** Periodical, Publishing, Research Personnel, Peer Review, General Surgery

## Abstract

**Purpose:**

To analyze the average time between submission and acceptance of national journals in seven Brazilian surgery journals from 2017 to 2022.

**Methods:**

It consists of a cross-sectional and observational study with a quantitative approach to analyze the acceptance time of articles approved by Brazilian journals on general surgery and its subspecialties, including *Acta Cirúrgica Brasileira, Jornal Vascular Brasileiro, Arquivos Brasileiros de Cirurgia de Digestiva, Revista do Colégio Brasileiro de Cirurgiões, Journal of Coloproctology, Revista Brasileira de Cirurgia Plástica*, and *International Brazilian Journal of Urology*.

**Results:**

The journals with the lowest average waiting times were *Revista do Colégio Brasileiro de Cirurgiões, Acta Cirúrgica Brasileira*, and *Journal of Coloproctology*, respectively, and, with the lowest interquartile range there is *Acta Cirúrgica Brasileira*. There was no significant difference between the pre-pandemic and pandemic periods. The study designs with the highest and lowest means were, respectively, ideas and innovations – also with the highest interquartile range – and expert opinion, while with the lowest interquartile range was technical skill.

**Conclusions:**

The acceptance time for articles in Brazilian surgery journals is extremely variable. Identifying these discrepancies highlights the importance of understanding editorial processes and seeking ways to improve consistency and efficiency in reviewing articles.

## Introduction

Scientific advancement in the medical field encompasses a set of developments that aim to improve the diagnosis, treatment, and prevention of various pathologies and health conditions. Through scientific research, technologies and medical techniques are developed, in addition to deepening knowledge in many areas, such as surgery^1^. Scientific advances have played an essential role in increasing the quality and expectation of life, contributing to reducing morbidity and mortality associated with various illnesses^2^.

In this context, the publication of scientific articles is one of the fundamental pillars for the advancement and dissemination of knowledge in medicine, including surgery. To achieve this, it is necessary to choose the appropriate journals, and it is essential to consider several factors, such as metrics. Through them, it is possible to evaluate the impact and relevance of journals, providing researchers with an important criterion to determine the best option for disseminating their research^3^.

Among these metrics, the time for acceptance of articles by journals stands out, which, ideally, should be as short as possible, since the scientific scenario evolves quickly. Such relevance is justifiable, as a long review time can make the results of a study obsolete even before they are published^4^. This can occur in the surgical setting, in which new techniques, approaches, and discoveries are constantly being developed. In this way, a short evaluation time allows data to be disseminated promptly, to promote the rapid updating of knowledge and the possibility of immediate application of discoveries in the medical field.

On the other hand, to avoid the publication and the consequent dissemination of lower-quality studies, susceptible to bias or misinterpretation, it is essential to submit this research to specialized reviews, preferably carried out by experts in the field. In this process, articles are critically analyzed by experts in the related field of study, called peer reviewers or expert reviewers. Peer review aims to ensure the quality and reliability of published scientific information, promoting integrity and the advancement of knowledge in the scientific community^5^.

In this context, this strategy aims to speed up the evaluation process, without compromising the necessary detail for a high-quality peer review^5^. According to Huisman and Smits^6^, the average time to complete the peer review process for articles in general categories is 17 weeks, with variations. It is worth noting that the medical field has one of the lowest average review durations^6^^–^ 8.

Therefore, it is observed that the publication of scientific articles plays a vital role in the dissemination of knowledge and in promoting the advancement of different areas of knowledge, a situation that is impacted by the time required for an article to be reviewed and accepted for publication. This period is variable, reflecting not only the complexities inherent to each discipline, but also the diversity of practices and processes adopted by different journals and publishers.

In the Brazilian scenario, this time variation is an issue that is still poorly documented, with scarce information, especially considering the context of surgery^9^. This gap is particularly relevant, considering the need to speed up the dissemination of results and advances in the surgical field. Based on this, this study aimed to analyze the average time between submission and acceptance of national journals in seven Brazilian surgery journals from 2017 to 2022.

## Methods

This is a cross-sectional and observational study with a quantitative approach, in which an analysis of the acceptance time of articles approved by Brazilian journals of general surgery and its subspecialties was carried out, from 2017 to 2022.

To this end, the submission date and acceptance date of all articles published and available in seven Brazilian surgery journals, related to general surgery and its subspecialties, were collected and included in the research, namely: *Acta Cirúrgica Brasileira, Jornal Vascular Brasileiro, Arquivos Brasileiros de Cirurgia de Digestiva, Revista do Colégio Brasileiro de Cirurgiões, Journal of Coloproctology, Revista Brasileira de Cirurgia Plástica*, and *International Brazilian Journal of Urology*.

Articles in which the submission and approval dates were not informed, as well as those that were removed from the journals or that were unavailable in the database, were excluded from the research.

For the analysis of the variables, the articles were classified according to acceptance time (TAC = difference between the date of submission and the date of acceptance, calculated in days) and category of type of study according to the classification of Scientific Electronic Library Online (SciELO): review article, special article, original article, letters to the editor, education, technical skill, ideas and innovations, clinical investigation, technical note, expert opinion, radiology page, case report, video section, technique, and others. Categories with fewer than 10 articles were grouped into “others,” including anesthesia, bioethics in surgery, statistician’s column, brief communication, prior note, scientific communication, and difference of opinion.

It is worth highlighting that some categories were grouped, as they have the same methodological design, namely: special article and featured article; surgical technique and technique; ideas and innovations, innovations, and biomedical technology; education, medical education, and teaching; and case report, challenging clinical cases, and therapeutic challenge. The journals that have publications in each category are organized according to Table 1.

**Table 1:** Scientific Electronic Library Online (SciELO) classification present in each journal in the analyzed period (2017–2022).

Review article	Special article	Original article	Letters to the editor	Challenging clinical case	Editorial comment	Therapeutic challenge	Education	Ideas and innovations
ABCD ACTA CBC JCOL JVB RBCP URO	ACTA JCOL RBCP	ABCD ACTA CBC JCOL JVB RBCP URO	ABCD CBC JCOL JVB RBCP URO	URO	URO	JVB	ACTA CBC	ACTA JVB RBCP
**Clinical investigation**	**Technical note**	**Expert opinion**	**Radiology page**	**Case report**	**Video section**	**Technique**	**Others**	
ACTA	CBC JCOL	URO	URO	JCOL JVB RBCP URO	URO	ABCD ACTA URO	ABCD ACTA URO	

ABCD: *Arquivos Brasileiros de Cirurgia Digestiv*a; ACTA: *Acta Cirúrgica Brasileira*; CBC: *Revista do Colégio Brasileiro de Cirurgiões*; JCOL: *Journal of Coloproctology*; JVB: *Jornal Vascular Brasileiro*; RBCP: *Revista Brasileira de Cirurgia Plástica*; URO: *International Brazilian Journal of Urology*. Source: Elaborated by the authors.

The data were organized and tabulated using Microsoft Word 2022 and Microsoft Excel 2022 softwares, and graphs derived from the analysis were generated. To construct boxplots, RStudio software version 4.2.1 was used. The absolute numbers of articles (N), and the means (M), quartiles (Q), and interquartile range (IR = Q3 - Q1) referring to TAC were considered according to the journal, SciELO category, and year of publication.

Regarding statistical analysis, the Statistical Package of the Social Sciences Statistics 20.0 program was chosen. To assess the presence or absence of a normal distribution, the Shapiro-Wilk’s test was used. Due to the variables not having a normal distribution, non-parametric statistics were used: the Kruskal-Wallis’ test to compare variables, and the Dwass-Steel-Critchlow-Fligner’s test to compare variables with each other. For comparative analysis between TAC and the pre-pandemic (2017 to 2019) and pandemic (2020 to 2022) years, the analysis of covariance (ANCOVA) test was used for the total TAC of all magazines by year, with Tukey’s post hoc for the TAC of individual magazines by years. *p* < 0.05 was considered significant and a 95%-confidence interval was adopted.

Considering that the interpreted data are present in public databases, it was not necessary to submit this study to the Research Ethics Committee.

## Results

From 2017 to 2022, 3,620 articles were analyzed from Brazilian journals on general surgery and its subspecialties. Considering all the journals, the minimum acceptance time interval varied significantly, with the minimum of one day and the maximum of 2,174 days (5.95 years). The average number of waiting days was 117.28, with an interquartile range of 79. The year 2017 recorded the longest waiting time (141.69 days), while 2020 recorded the shortest one (103.47 days) (Table 2).

**Table 2:** Article acceptance time per year and per journal in the evaluated period (2017–2022). ANCOVA TAC pre-pandemic × pandemic (*p* = 0.056); Tukey’s post hoc test TAC pre-pandemic × pandemic CBC (*p* = 0.006); Tukey’s post hoc test TAC pre-pandemic × pandemic URO (*p* < 0.001); Kruskal-Wallis’ test TAC all journals (*p* < 0.001); Kruskal-Wallis’ test TAC CBC × ACTA × JCOL (*p* < 0.001).

Year	CBC					ACTA					JVB			
	M	Q1	Q3	N		M	Q1	Q3	N		M	Q1	Q3	N
2017	58.63	37	73	86		90.90	91	95	115		93.11	61	119.25	54
2018	56.18	33.25	73	78		87.31	88.25	93	118		131.55	89.75	152	56
2019	48.36	30	62	70		93.37	87	91	101		104.77	61.5	129.2	56
2020	53.61	20	71	88		89.57	88	91	94		116.04	57	164.5	74
2021	86.62	40	111.50	63		89.79	88	92	90		93.03	55.25	116.75	98
2022	144.05	60	212	81		85.51	86	91	83		132.55	89	164	55
**Total**	**74.36**	**33**	**84**	**466**		**89.66**	**88**	**93**	**601**		**109.96**	**65**	**145**	**393**
**Year**	**ABCD**					**JCOL**					**RBCP**			
	**M**	**Q1**	**Q3**	**N**		**M**	**Q1**	**Q3**	**N**		**M**	**Q1**	**Q3**	**N**
2017	114.47	82	120.5	70		84.67	30.5	107.5	58		226.66	89	266	97
2018	76.47	55.25	69	70		78.00	40.5	96.5	58		284.82	104	317.2	76
2019	116.35	69	120.5	72		87.93	46	105	61		155.81	80.25	220.5	88
2020	93.33	74.75	109.75	72		49.38	28	66	69		192.92	77	274.5	83
2021	109.87	80	116	77		109.63	49	157.8	72		217.50	120.2	297.5	78
2022	120.54	50 25	152	74		103.82	64	124	57		182.52	86.5	234	79
**Total**	**110.47**	**65**	**113**	**435**		**85.38**	**39**	**120**	**375**		**209.06**	**88**	**266**	**501**
**Year**	**URO**					**Total**								
	**M**	**Q1**	**Q3**	**N**		**M**	**Q1**	**Q3**	**N**					
2017	219.35	112.2	275.5	162		141.69	72	172.8	642					
2018	127.51	72	167	181		120.34	61	133	637					
2019	129.94	63	182.8	164		110.38	58	146	612					
2020	106.11	35	158	112		103.47	46	116.5	592					
2021	90.18	12.5	122	131		111.25	55	133	609					
2022	38.91	6	62	99		113.18	53	136	528					
**Total**	**127.29**	**46**	**185**	**849**		**117.28**	**58**	**137**	**3620**					

ABCD: *Arquivos Brasileiros de Cirurgia Digestiva*; ACTA: *Acta Cirúrgica Brasileira*; CBC: *Revista do Colégio Brasileiro de Cirurgiões*; JCOL: *Journal of Coloproctology*; JVB: *Jornal Vascular Brasileiro*; RBCP: *Revista Brasileira de Cirurgia Plástica*; URO: *International Brazilian Journal of Urology*; N: numbers of articles; M: mean; Q: quartil; IR: interquartile range.Source: Elaborated by the authors.

When comparing the pre-pandemic periods from 2017 to 2019 (M = 121.41 days) and the pandemic periods from 2020 to 2022 (M = 109.17 days) (Table 2), no significant difference was observed between the periods (*p* = 0.056) according to the ANCOVA test. However, when analyzing individually, only two journals showed a statistical difference between the two periods: *Revista do Colégio Brasileiro de Cirurgiões* (*p* = 0.006) and *International Brazilian Journal of Urology* (*p* < 0.001), according to Tukey’s post hoc test.

**Figure 1:**
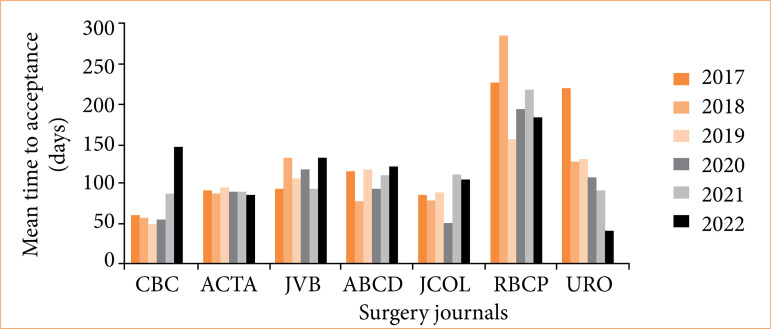
Mean of time to acceptance in days per journal and per year during the evaluated period (2017–2022). Kruskal-Wallis’ test TAC all journals (*p* < 0.001).

CBC: *Revista do Colégio Brasileiro de Cirurgiões*; ACTA: *Acta Cirúrgica Brasileira*; JVB: *Jornal Vascular Brasileiro*; ABCD: *Arquivos Brasileiros de Cirurgia Digestiva*; JCOL: *Journal of Coloproctology*; RBCP: *Revista Brasileira de Cirurgia Plástica*; URO: *International Brazilian Journal of Urology*. Source: Elaborated by the authors.

**Figure 2:**
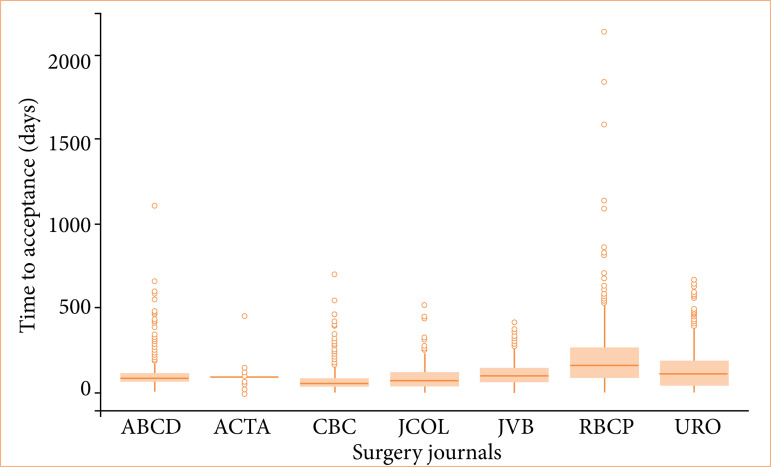
Box-plot graphs related to time of acceptance in days per journal during the evaluated period (2017–2022). Kruskal-Wallis’ test TAC all journals (*p* < 0.001).

CBC: *Revista do Colégio Brasileiro de Cirurgiões*; ACTA: *Acta Cirúrgica Brasileira*; JVB: *Jornal Vascular Brasileiro*; ABCD: *Arquivos Brasileiros de Cirurgia Digestiva*; JCOL: *Journal of Coloproctology*; RBCP: *Revista Brasileira de Cirurgia Plástica*; URO: *International Brazilian Journal of Urology*. Source: Elaborated by the authors.

The journal with the lowest average acceptance time, considering all years studied, was the *Revista do Colégio Brasileiro de Cirurgiões* (74.36 days), with the total of 466 publications. On the other hand, the *Revista Brasileira de Cirurgia Plástica* exhibited the highest average TAC (209.06 days) and interquartile range (178 days), totaling 501 publications (Table 2 and Figs. 1 and 2).

Regarding the number of publications, the largest was from the *International Brazilian Journal of Urology*, with 849 articles, and the smallest was from the *Journal of Coloproctology*, with 375 (Table 2 and Figs. 1 and 2). The Kruskal-Wallis’ test revealed statistically significant differences in the TAC between all the journals (*p* < 0.001), as well as between the Dwass-Steel-Critchlow-Fligner’s multiple comparisons of the different journals among themselves.

**Figure 3:**
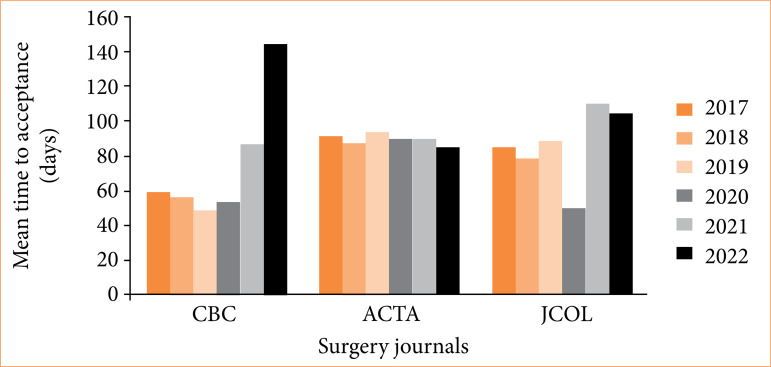
The three journals with the best average acceptance times in days during the analyzed period (2017–2022). Kruskal-Wallis’ test TAC CBC × ACTA × JCOL (*p* < 0.001).

CBC: *Revista do Colégio Brasileiro de Cirurgiões*; ACTA: *Acta Cirúrgica Brasileira*; JCOL: *Journal of Coloproctology*. Source: Elaborated by the authors.

Between the three journals with the lowest TAC averages, despite the *Revista do Colégio Brasileiro de Cirurgiões* having maintained the lowest values for most of the analyzed period, in the years 2020 and 2022, the *Journal of Coloproctology* (49.38) and *Acta Cirúrgica Brasileira* (85.51) had shorter waits, respectively (Table 2 and Fig. 3).

Furthermore, it is noteworthy that *Acta Cirúrgica Brasileira* presented the greatest stability in waiting time, a fact evidenced by the lowest interquartile range (IR = 5 days) and one of the lowest total averages (89.66 days), with 601 articles published (Table 2 and Figs. 1, 2, and 3).

Kruskal-Wallis’ test showed statistically significant differences in the TAC between all the three journals (*p* < 0.001) and between the Dwass-Steel-Critchlow-Fligner’s multiple comparisons of the three journals among themselves.

**Figure 4:**
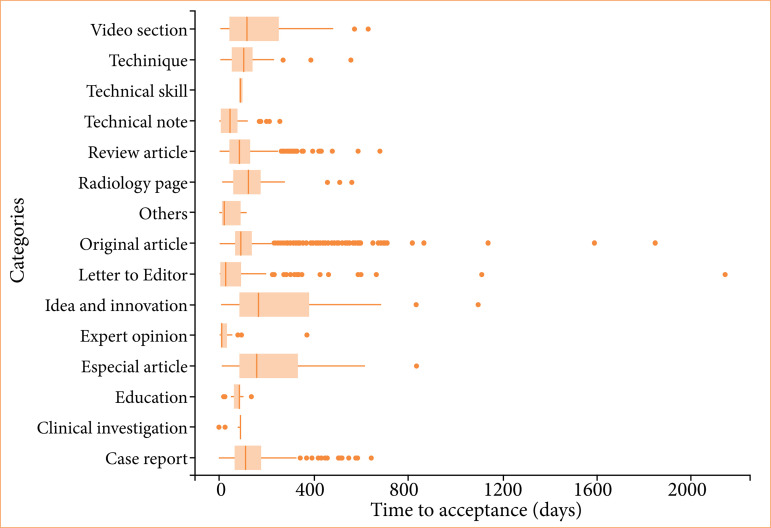
Box-plot graph related to time of acceptance in days per categories from Scientific Electronic Library Online (SciELO) during the evaluated period (2017–2022). Kruskal-Wallis’ test TAC all categories (*p* < 0.001).

Source: Elaborated by the authors.

About the totality of magazines analyzed, the type of study with the highest average TAC and interquartile range was ideas and innovations (M = 266.54; IR = 290), with 35 articles. In turn, the lowest average was in expert opinion (28.32), with 41 studies, and the lowest interquartile range was in technical skill (M = 88.31; IR = 4), with 13 publications (Table 3 and Fig. 4). There were statistically significant differences in the TAC between the categories according to the Kruskal-Wallis’ test, as well as between the Dwass-Steel-Critchlow-Fligner’s multiple comparisons of the different classes of articles among themselves.

**Table 3:** Time to acceptance in days per categories from Scientific Electronic Library Online (SciELO) during the evaluated period (2017–2022). Kruskal-Wallis’ test TAC all categories (*p* < 0.001).

Categories	M	Q1	Q3	N
Review article	100.73	43.5	127.5	359
Special article	214.97	87.5	331	34
Original article	117.47	68	133	2320
Letters to the editor	107.57	6	87.75	130
Education	75.1	63	89	20
Technical skill	88.31	87	91	13
Ideas and innovations	266.54	87.5	377.5	35
Clinical investigation	85.34	87	92	35
Technical note	57.71	8.5	68	42
Expert opinion	28.32	7	28	41
Radiology page	44.43	59	175	30
Case report	150.78	66.5	174.5	37
Video section	130.20	48.25	251.25	367
Technique	152	56	135.5	94
Others	111.49	13.5	84.75	63
Total	117.28	58	137	3620

M: Mean; Q1: Quartile 1; Q3: Quartile 3; N: Number of articles published. Source: Elaborated by the authors.

## Discussion

The identification of journals with long or short acceptance times can be extremely relevant both for authors, who face crucial decisions in choosing the publication vehicle for their scientific work, and for the journals themselves, which constantly seek to improve and optimize their processes related to review and publication^10^. This aspect assumes even greater importance in a constantly evolving academic and scientific context, in which efficiency in the dissemination of knowledge plays a fundamental role^11^.

The results revealed a wide variation in the acceptance times of articles in scientific journals of surgery in the period from 2017 to 2022. Notably, some journals present low averages and a reduced interquartile range in the time required for acceptance, which suggests the efficiency of its review and approval processes. On the other hand, the maximum time of 2,174 days (equivalent to 5.95 years) highlights significant challenges in the editorial process of some publications. The average waiting days of 116.70, with an interquartile range of 79 for the 3,620 accepted articles, indicate a considerable dispersion in response times to authors, reflecting the heterogeneity in the editorial rigor and workload of the journals.

Furthermore, analysis by journal revealed notable discrepancies in the average acceptance time, with the *Revista Brasileira de Cirurgia Plástica* demonstrating the highest average acceptance time and the largest interquartile range, suggesting variability in its review procedures. On the other hand, among the three journals that stood out in terms of effectiveness, it was observed that *Acta Cirúrgica Brasileira* achieved the greatest stability in its evaluation, as it had a smaller interquartile range compared to the others.

In comparison to foreign studies, in a British analysis of plastic surgery journals, the median acceptance time was 4.6 months (138 days)^11^, revealing a considerable time for approval, but even shorter when compared to the Brazilian journals from the same area. In addition, the *British Journal of Surgery* obtained an average time between manuscript submission and the first decision on the article of 22 days^12^, being able to maintain the quality of the review in a relatively short evaluation period. Concerning medical journals with general clinical themes, Sebo (2022)^13^ highlights that the median acceptance of articles published in different countries between 2012 and 2022 was 68 days, with progression over the years, resulting in an average time of 49 days in 2022. In this scenario, it is observed that Brazilian surgery journals are still at a disadvantage.

Furthermore, a shorter acceptance period contributes to improving the authors’ quality of life, reducing levels of anxiety and stress, which is a relevant aspect, given that medical professionals and students in the field face significantly higher mental loads than the general population^14^. In this sense, journals that presented a low interquartile range allow the researcher to estimate the time needed to wait for the verdict on their article. On the other hand, journals with a larger interquartile range can make it difficult to plan deadlines for presenting their theses, with the possibility of further affecting mental health, especially among postgraduate students in the field of medicine, who are charged with scientific publications following certain deadlines^15^^–^ 17.

Unlike book publication scenarios that occur later, articles have the characteristic of being quickly disseminated and, as a result, there is some pressure to speed up the review process coming mainly from the scientific community, specifically, from the authors^4^^,^ 9. The findings of the present study revealed that each journal has its specific ways of working, and, therefore, there is variability in average acceptance times. In this context, the *Acta Cirúrgica Brasileira* had the best result in this regard, as a lower interquartile range indicates standardization of peer review and editorial analysis procedures, as well as rigor regarding deadlines.

In this context, medical literature requires a rush for publication, so the more outdated it becomes, the lower the chances of acceptance by journals. This situation is aggravated, mainly, when research is a continuation of other works and, thus, becomes hostage to the acceptance time of the previous study for the publication itself, or even when the journal does not send its evaluation and the authors are unable to submit the article to other journals^18^. The more serious this scenario is, the less the stimulus to write becomes, thus configuring an obstacle to the development of scientific knowledge on national soil^16^.

It should be noticed that there is a relationship between a good quality review by scientific journals and the time invested in this activity; however, there is a kind of plateau, which means that, after a certain time, the increase in time is not related to a higher quality of the review^19^. Therefore, the evaluation must be carried out in detail to avoid the publication of biased articles, with biases or erroneous interpretations in the shortest possible time without affecting the excellence of the process.

To avoid conflicts of interest, the ideal is to carry out the peer review process made up of experts in the field^6^^,^ 20. In addition, there is the fact that the position of a reviewer is voluntary, so reviewers are not paid for the time spent, and knowing that not only the occupation of a medical doctor but even more so that of a surgeon has a very high workload makes it even more delicate to demand speedy review^6^^,^ 21. For these reasons, reviews must be carried out in the shortest possible time, but respecting the need to be carried out with caution, and attention to detail, and done by experts on the subject, becoming one of the biggest challenges for journals and reviewers to balance between agility and careful analysis.

Regarding an ideal time as recommended by medical journals, the International Committee of Medical Journal Editors does not suggest a specific interval due to the diversity of study types, which reflects the variability of acceptance times. However, such an organization reinforces the need for quick and timely processing of manuscripts according to available resources^22^. Furthermore, in the Zabala et al.’s^7^ study, the comparison between countries related to evaluation days and publication days revealed that Brazil is below the expected average, meaning a longer delay, which points out the need for alternatives to improve current performance, although the medicine area is one of the most efficient^6^.

Concerning the years 2020 to 2022, during which a delay in the publication of numerous journals was expected due to the COVID-19 pandemic – scientists presented difficulties related to the new context due to the reduction of editorial capacity along with the high demand for work in hospitals –, there was no significant difference when compared to the other years analyzed^19^. This lack of influence may be a consequence of the review work taking place mostly online and, due to the change in routine, such as in cases of cancellation of elective surgeries caused by social isolation, so there would be more time to correct studies and, thus, contribute to the non-change or reduction of the TAC^23^. This hypothesis differs from the reality of journals directly related to the topic of COVID-19, in which there was a significantly greater number of publications as preprints, together with the speed of publications on discoveries linked to the pandemic^22^^,^ 24.

Another factor that can influence the TAC refers to the types of study, as experimental research or clinical trials have greater ethical rigor compared to observational studies or articles on expert opinion. Added to this, studies in which the authors are editors or renowned authors take less time compared to studies by inexperienced and unguided researchers. This is also reflected in expert opinion articles, which are often produced by just one author, who is invited to speak on the subject in thematic editions of the magazine, with the magazine being expected to correct more easily and in less time^25^. In this sense, the results of the present study revealed a proportional relationship between the TAC and the level of complexity of the methodological design, since primary studies require more methodological care from reviewers compared to secondary studies.

Another relevant issue is the professionals’ quality of life , which is negatively affected by the set of difficulties that the country faces with little investment in science and technology and with a still incipient research infrastructure^9^. This occurs mainly because the reviewers’ work is not properly recognized by the scientific community, coupled with the difficulty in finding a minimum number of qualified reviewers who respond to requests for opinions promptly, to avoid delays in publication of articles^9^^,^ 26.

The main limitations of this study are related to the possibility of errors in the classification of articles in different journals, as well as potential inaccuracies in the dates of the articles, due to data collection being carried out manually. However, it is important to highlight that these potential sources of error were mitigated through verification by at least two independent researchers of these classifications and values, to avoid possible errors.

Furthermore, it is important to mention that data collection from editorials could not be done due to the lack of acceptance dates. In addition, blocked articles were found due to publication in more than one journal. Their inclusion in the present study was unfeasible, being a limitation referring to the lack of data.

In this scenario, it is observed that the results obtained offer insights and valuable information that can be analyzed in the context of improving editorial processes in medical journals, especially surgical journals, thus contributing to improving the evaluation process of scientific journals in this area. Finally, the present study can support new goals formulated by the journals, as well as suggests studies with a similar methodology applied to other areas, mainly in Brazil, due to the presentation of issues addressed especially in the country and which may be a reality in other journals, a perspective still little addressed.

## Conclusion

The results of the present study showed that the acceptance time for articles in Brazilian surgery journals is extremely variable. This oscillation can be explained by several factors, such as the type of study, the journal, the number of authors and reviewers involved, the complexity of the work, and the existence of conflicts of interest.

Furthermore, identifying these discrepancies in acceptance times highlights the importance of understanding editorial processes and seeking ways to improve consistency and efficiency in article review. Such results can be fundamental for improving editorial procedures, benefiting the scientific community, and minimizing authors’ anxiety associated with publication deadlines.

## Data Availability

All dataset were generated or analyzed in the current study.
